# 

**Published:** 1869-06

**Authors:** 


### CONTENTS.

### ORIGINAL COMMUNICATIONS.

Dental Hygiene .......... 229
Health of Dentists .......... 235
Contour Fillings .......... 237
The Hands .......... 242
Operative Dentistry .......... 243

### PROCEEDINGS OF SOCIETIES.

Northern Ohio Dental Association .......... 245
Forest City Society of Dental Surgeons .......... 250

### SELECTIONS.

The Administration of Chloroform .......... 252
Alveolar Abscess .......... 253
Anæsthetics Adverse to Union by First Intention .......... 253
Chloroform Inhalations in Strychnia Poisoning .......... 253
Iodine and Aconite in Periodontitis .......... 254
New Uses for Mica .......... 254
Assimilation of Phosphate of Lime and its Therapeutical Employment .......... 254
Alcohol as a Dressing to Wounds .......... 255
Hypophosphites in the Tooth-ache of Pregnancy .......... 255
Classification of Boston Doctors .......... 256
A New Growing Slide .......... 256
On the Use of Chloride of Gold in Microscopy .......... 257
New Mode of Preparing Objects for the Microscope .......... 258
The Frst Physician in Mssachusetts .......... 258
Treatment of Diseased Gums .......... 259
Hypodermic Method of Injection .......... 259
Japanese Dentistry .......... 262
Death from Hypodermic Injection .......... 263

### EDITORIAL.

Calcification of Tooth Pulp .......... 264
A Speech .......... 265
Another Rubber Patent .......... 268
Suspension of the N. Y. College of Dentistry .......... 270
The Boston Dental College .......... 271
Supreme Judicial Court .......... 271

